# Stability of Extracellular Vesicle Composition in Blood Specimens: Impact of Pre‐analytical Factors and Isolation Methods

**DOI:** 10.1002/alz70856_105924

**Published:** 2026-01-10

**Authors:** Eunju Seong

**Affiliations:** ^1^ Streck LLC, La Vista, NE, USA

## Abstract

**Background:**

Extracellular vesicles (EVs) circulating in blood hold significant diagnostic potential for various diseases, including neurological disorders. However, as a living tissue, blood undergoes substantial changes during ex vivo transition and specimen handling. Pre‐analytical factors, such as the choice of blood collection tubes and blood processing methods, can activate blood cells (e.g., platelets, neutrophils, monocytes), potentially altering EV composition. To assess the effects of these pre‐analytical factors on the stability of EV composition in blood specimens, we examined EV populations in plasma samples from healthy donors following various collection and processing methods.

**Method:**

MACSPlex EV Kits were used to profile multiple EV populations simultaneously in a single sample via flow cytometry. Using Immuno‐Oncology and Neuro EV panels, we evaluated 63 EV populations in blood collected in three types of tubes: EDTA, ACD‐A and Protein Plus BCT, processed at various times. We also paired these assays with three EV isolation methods, including precipitation and size exclusion chromatography, to assess the impact of EV enrichment on EV profiling.

**Result:**

When plasma isolation was delayed, certain EV populations in blood increased significantly during storage in both EDTA and ACD‐A tubes, particularly those labeled with surface markers related to immune response and inflammation (e.g., CD9, CD14, CD24, CD29, CD45) and activated platelets (e.g., CD42a, CD62P). Conversely, the overall EV profile in Protein Plus BCT remained close to the draw‐time composition. EV isolation methods enhanced EV binding to capture beads, enabling more reliable measurement of circulating EVs associated with the nervous system (e.g., GLAST, PSA‐NCAM, VGLUT2).

**Conclusion:**

Our findings demonstrate that EV composition remains stable in Protein Plus BCT compared to EDTA and ACD‐A tubes, with a small number of exceptions. This insight into EV stability in blood specimens is crucial for advancing EV biomarker research in neurological disorders, including Alzheimer's disease, potentially enhancing diagnostic accuracy and therapeutic strategies.

